# A Retrospective Analysis of Conservative Management Versus Early Surgical Intervention in Appendicular Lump

**DOI:** 10.7759/cureus.21784

**Published:** 2022-01-31

**Authors:** Bhupati Bhusan Das, Kedar Nath Nayak, Sujit Kumar Mohanty, Ashok Kumar Sahoo

**Affiliations:** 1 Surgery, Saheed Laxman Nayak Medical College and Hospital, Koraput, IND; 2 Surgery, Bhima Bhoi Medical College and Hospital, Balangir, IND; 3 Surgery, Srirama Chandra Bhanja Medical College and Hospital, Cuttack, IND; 4 Surgery, Jawaharlal Institute of Postgraduate Medical Education & Research, Puducherry, IND

**Keywords:** luminal obstruction, early exploration, interval appendectomy, ochsner-sherren regime, appendicular lump

## Abstract

Introduction

Acute appendicitis happens to be increasingly common in school-going children and early part of adult life, peak incidence reaching in the teens and early twenties. Luminal obstruction of the vermiform appendix is thought to be essential for the development of appendicular ischemia, gangrene, and perforation. The treatment of choice in acute appendicitis is emergency appendectomy. Appendicitis particularly puts the surgeon in a dilemma whenever the patient presents late by around four to seven days. In case of delay in presentation, complications like appendicular lump formation occur. The factors that make the clinical presentation inconsistent which in turn makes the diagnosis challenging in the case of acute appendicitis are the variable position of the appendix, the degree/grade of inflammation, and the age of the patient.

At present, the standard treatment is the Ochsner-Sherren regimen universalized by Oschner and has been mostly practised over many decades as the standard care for the appendicular lump. Conservative regimen does not work in a few cases where urgent surgical exploration is necessary. This study was conducted to compare early appendectomy versus conservative management followed by interval appendectomy in case of appendicular mass and to make a better strategy for effective management of patients with complicated appendicitis.

Methods

A total of 112 patients were diagnosed as having an appendicular lump as per the available records between June 2018 and June 2021. The total study population was divided into two comparative groups depending upon the treatment they received. The patients in group-1 received medical treatment and those in group-2 had undergone surgical management. The patients in group-1 were treated according to the Ochsner-Sherren regimen. The patients in group-2 were the patients in whom emergency appendectomy was done. If the general condition of the patient did not improve, pain and tenderness didn’t subside, the size of phlegmon or abscess was increasing and other features of the acute abdomen were persistent, then it was regarded as a failure of medical treatment and the patient was prepared for surgery on an emergency basis.

Results

Out of 1192 cases of acute appendicitis admitted between June 2018 and June 2021, a total of 112 patients were diagnosed with an appendicular lump. As per the record, 64 patients were managed conventionally as per the Ochsner-Sherren regimen followed by elective interval appendectomy (group-1) and 48 cases were managed with an emergency surgical procedure (group-2).

In group-1, out of 64 patients, non-operative treatment was successful in 58 patients (90.62%). Among the remaining patients, there was a failure of non-operative treatment in six patients and they were subjected to emergency surgical exploration (9.37%). So a total of 58 patients underwent interval appendectomy after six weeks. Out of 54 patients who had undergone emergency appendectomy in both groups, the per-operative finding was an appendicular lump in 55.5% of patients while a total of 44 patients in group-2, were discharged from the hospital within six days (91.66%). But in group-1, only 16 patients were discharged from the hospital within six days (25%), rest were discharged from the hospital after more than seven days of stay.

Conclusion

Early appendectomy in appendicular mass is safe due to the improvements in surgical techniques and better postoperative care.

## Introduction

Acute appendicitis is one of the most common surgical conditions of acute abdomen. The significance of the vermiform appendix for a surgeon results primarily from its tendency for inflammation, which results in the clinical condition called acute appendicitis. This clinical entity appendicitis is so common that appendectomy is the most commonly performed emergency abdominal operation. Appendectomy is often the first major procedure performed by a surgeon in training. Besides its propensity to cause surgical pathology, the vermiform appendix, long thought to be a vestigial organ, is believed to have important roles in immune function and maintaining the gut microflora [1]. Acute appendicitis is very rare in infants. Acute appendicitis happens to be increasingly common in school-going children and early part of adult life, peak incidence reaching in the teens and early twenties [2].

Luminal obstruction of the vermiform appendix is thought to be essential for the development of appendicular ischemia, gangrene, and perforation [3]. But in many cases, it is seen that the appendicular lumen is usually patent in the early stages of appendicitis, with the presence of mucosal inflammation and lymphoid hyperplasia. Mostly the cause of luminal obstruction is a faecolith, also called an appendicolith. It is composed of inspissated fecal material, bacteria, calcium phosphates, and epithelial debris. Rarely, a foreign body is incorporated into the fecolith. It is to note that incidental finding of a fecolith is a relative indication for a prophylactic appendectomy or an interval appendectomy in a patient treated conservatively [4].

The lifetime risk of appendicitis is 8.6% among males and 6.7% among female persons [5]. But in contrast, the risk of undergoing appendectomy depicts an opposite trend; with the female gender undergoing appendectomy in their lifetime is approximate twice as many as that of male patients [6]. The treatment of choice in acute appendicitis is emergency appendectomy. Appendicitis particularly puts the surgeon in a dilemma whenever the patient presents late by around four to seven days. In case of delay in presentation, complications like appendicular lump formation occur [7].

Timely diagnosing a case of acute appendicitis is clinically challenging especially when there is atypical presentation. These cases usually get missed at the emergency department. The statistical report reveals that one in five cases of appendicitis are misdiagnosed and a normal appendix is detected in 15% to 40% of cases who have undergone appendectomy in emergency surgery [8]. The factors that make the clinical presentation inconsistent which makes the diagnosis challenging in the case of acute appendicitis are the variable position of the appendix, the degree/grade of inflammation, and the age of the patient. The appendicular base is fairly constant in position at the posteromedial wall of the cecum where the three taeniae converge. But the location of the tip varies greatly with pelvic in 55.8%, subcecal in 19%, retroileal in 12.5%, retrocecal in 7%, ectopic in 4.2%, and preileal in 1.5% of cases [9].

An appendicular lump is a term/condition in which there is an inflammatory mass consisting of the inflamed appendix, greater omentum, adjacent ileum, cecum, and ascending colon sometimes containing a variable amount of pus. Appendicular lump is usually seen after 48 to 72 hours of the onset of symptoms of acute appendicitis. Delayed diagnosis converts the uncomplicated simple acute appendicitis into complicated appendicitis. It is observed that appendicular lump is seen in 2% to 6% of cases of acute appendicitis if emergency surgery is not done [10]. The appendicular lump is usually witnessed amongst elderly males [11].

Classical teaching is that the appendicular lump should not be operated on and to be managed according to the Ochsner-Sherren regime. This strategy of the regime is based on the presumption that the inflammatory process is already localized and that inadvertent surgery is challenging and may be unsafe. It may be difficult to identify the appendix and occasionally due to inadvertent injury, a fecal fistula formation may develop. For the above-said reasons, it is wise to undertake non-operative management. At the same time one must be prepared for surgery should clinical deterioration occur. At present, the standard treatment is the Ochsner-Sherren regimen universalized by Oschner and has been mostly practised over many decades as the standard care for the appendicular lump [12]. Conservative regimen does not work in 2% to 4% of cases (up to 10% of cases), where urgent surgical exploration is necessary [13]. Ochsner-Sherren's regime is favoured mainly because it can avoid potential complications like cecal perforation and the occurrence of fecal fistula [14]. The success rate of the Ochsner-Sherren regimen is about 88% to 95% [15]. Since there is a high chance of recurrence of appendicitis and formation of appendicular lump following Ochsner-Sherren management, interval appendectomy is mandatory [16]. Other pathological conditions like malignancy of cecum and ileocaecal tuberculosis can mimic acute appendicitis [17]. In the present modern era where resources and expertise for laparoscopic surgery and anaesthesia equipment are available, early exploration of appendicular lumps is advised, which minimizes the hospital stay, is curative and helps in diagnosing the disease, and prevents the need for second hospital admission without an increase in morbidity and mortality. Hence the study was conducted to compare early appendectomy versus conservative management followed by interval appendectomy in case of appendicular mass and to make a better strategy for effective management of patients with complicated appendicitis.

## Materials and methods

We systemically searched the hospital database for new diagnoses of acute appendicitis in a tertiary care hospital in Eastern India between June 2018 and June 2021. A complicated appendicitis was defined as having features of appendicitis, terminal ileitis, typhlitis, omental adhesions, with or without abscess often forming a lump [18]. A total of 112 patients were diagnosed as having an appendicular lump in those three years according to the available records. All the patients of both genders and any age group with clinical findings suggestive of an appendicular lump or those patients who were diagnosed as having appendicular lump during emergency surgery for acute appendicitis were included in the study. The patients who were excluded from the study were pregnant ladies, patients with diffuse peritonitis and septicaemia, those whose diagnosis changed following surgery or the patients who lost scheduled follow-up. A detailed history was taken along with a thorough clinical examination for every patient. Laboratory parameters like completed blood counts, erythrocyte sedimentation rate (ESR), random/fasting blood sugar, serum urea and creatinine, serum sodium and potassium were done. Viral markers like human immunodeficiency virus (HIV), hepatitis C virus (HCV), and hepatitis B surface antigen (HBsAg) were done. To confirm the diagnosis, ultrasonography (USG) of the abdomen was done in all cases. The findings of bowel wall thickening, echogenic mesentery, and free fluid with features of appendicitis indicated underlying complicated appendicitis. In cases of doubt regarding the diagnoses, a contrast-enhanced computed tomography (CECT) scan of the abdomen was ordered. Along with the above USG findings with contrast enhancement of the wall and lack of contrast filling, fat stranding had pointed towards complicated appendicitis.

The total study population was divided into two comparative groups depending upon the treatment they received. The patients in group-1 received medical treatment and those in group-2 had undergone surgical management. The patients in group-1 were treated according to the Ochsner-Sherren regimen [12]. According to the regimen, the patients were kept nil per orally, and were started on broad-spectrum antibiotics, analgesics and intravenous fluids till the acute symptoms subsided, followed by interval appendectomy. The term interval appendectomy was defined as the surgery was done after six weeks of the completion of conservative management. The patients in group-2 were the patients in whom emergency appendectomy was done. If the general condition of the patient did not improve, pain and tenderness didn’t subside, the size of phlegmon or abscess was increasing and other features of acute abdomen were persistent, then it was regarded as a failure of medical treatment and the patient was prepped for surgery on an emergency basis. For the patients in whom the surgery was done within 24 hours of hospitalization, it was defined as early appendectomy [19].

For one month after surgery (first, second and fourth week of surgery) after appendectomy (early or interval) and at first, the second and fourth week of conservative management was the intended follow-up.

Data were analysed using Microsoft Office Excel 2007 (Microsoft, Washington, USA) and Statistical Package for the Social Sciences (SPSS, version 16.0, SPSS Inc., Chicago, Illinois, USA) software. The data were expressed as frequencies and percentages. The Chi-square test was used to study the relationship between categorical variables. A p-value of less than 0.05 was considered statistically significant.

## Results

Out of 1192 cases of acute appendicitis admitted over the said period of time, a total of 112 patients were diagnosed with an appendicular lump and were selected based on inclusion criteria. According to the demographic data, most of the patients belonged to the age group of 21 to 30 years (Table 1).

**Table 1:** Age distribution of patients

Age in Years	Number (n)	Percentage (%)
Up to 10	0	0
11 to 20	26	23.21 %
21 to 30	40	35.71 %
31 to 40	32	28.57 %
41 to 50	6	5.35 %
51 to 60	4	3.57 %
Above 6o	4	3.57 %

Both the groups had equal gender distribution (Table 2).

**Table 2:** Gender distribution of patients

Gender	Group 1		Group 2	
Male	42	65.62%	30	62.5%
Female	22	34.37%	18	37.5%

In both groups, the number of male patients was higher. Most of the patients were admitted to the hospital after the fourth day of onset of pain in both the groups (Table 3).

**Table 3:** Time between onset of pain and admission to hospital

Time in Days	Group 1		Group 2		Total	
	Number	Percentage	Number	Percentage	Number	Percentage
≤2	4	6.25%	4	8.33%	8	7.14%
3	20	31.25%	12	25.0%	32	28.57%
4	24	37.5%	20	41.66%	44	39.28%
5	14	21.87%	10	20.83%	24	21.42%
≥6	2	3.12%	2	4.16%	4	3.57%

The number of admissions after the fourth day of onset of pain was 24 and 20 in groups 1 and 2 respectively.

As per the record, 64 patients were managed conventionally as per the Ochsner-Sherren regimen followed by elective interval appendectomy (group-1) and 48 cases were managed with an emergency surgical procedure (group-2).

In group-1, out of 64 patients, non-operative treatment was successful in 58 patients (90.62%). Among the remaining patients, there was a failure of non-operative treatment in six patients and they were subjected to emergency surgical exploration (9.37%). So a total of 58 patients underwent interval appendectomy after six weeks. One patient from group-2 developed fecal fistulas and the patient was successfully managed conservatively. Out of 54 patients who underwent an emergency appendectomy in both the groups, the per-operative finding was an appendicular lump in 55.5% of patients (Table 4).

**Table 4:** Per operative findings

Operative Finding	Number	Percentage
Appendicular lump	30	55.5%
Gangrenous appendicitis	16	29.6%
Appendicular perforation with abscess	8	14.8%

The rest of the patients had either gangrenous appendicitis (29.6%) or appendicular abscess with perforation (14.8%).

A total of 44 patients in group-2, were discharged from the hospital within six days (91.66%) (Table 5).

**Table 5:** Duration of hospital stay

Hospital Stay	Group 1	Group 2	Total	p-value
≤ 3 days	0	28 (58.33%)	28 (29.16 %)	0.00012
4 to 6 days	16 (25%)	16(33.33%)	32 (28.57%)	
≥7 days	48 (75%)	4 (8.33%)	52 (46.42%)	

The majority of the patients (58.33%) were discharged from the hospital within three days of admission. But in group-1, only 16 patients were discharged from the hospital within six days (25%), and the rest were discharged from the hospital after more than seven days of stay.

## Discussion

Appendicular lump also called phlegmon is mostly formed after an acute attack of appendicitis, which is mostly seen as a palpable tender mass in the right iliac fossa. It ranges from a lump/mass/phlegmon and in worst cases, abscess formation [20,21]. In the present study, the incidence of the appendicular mass was 9.39%, which is equivalent to studies of other authors [11-13,22]. A Nigerian study found the incidence of 2.9% while for Bhandari et al. it was 2% to 6%. The majority of cases were found to be in the age group of 21 to 30 years (35.71%) and the gender ratio of male:female was around 2:1, which is similar to other studies [21,23]. A study by Pandey et al. revealed the majority of the patients (48.39%) from the age group of 21 to 30 years with male:female was approximately 2:1 [21]. Similarly, another study from Northern India showed 41.3% were from the age group of 21 to 30 years while male and female patients were 31 and 15 respectively [23]. A total of 44 patients (39.28%) had pain in the right iliac fossa for four days. However, the commencement of right lower quadrant pain ranged between two to 15 days. Conservative treatment failed in six patients in group-1 and they underwent immediate emergency exploration. An intra-operative finding of the appendicular lump was seen in 55.5% of patients and gangrenous appendicitis was seen in 29.6% of patients whereas appendicular perforation with abscess was seen in 14.8% of patients and is comparable to other literature [24,25]. In group-2, patients treated with the early surgical exploration of appendicular mass, hospital stay was reduced to three days in 58.33% of cases, four to six days in 33.33% of cases, and a single patient (4.16%) developed faecal fistula who required a hospital stay of more than two weeks and was managed conservatively with success. Malik et al. documented perforated appendix, loculated collection of pus, appendicular abscess in eight, seven, and four patients, respectively, in the early surgery group whereas these cases were nil in the interval appendectomy group [24]. Patel et al. in their study found that 56% of patients had appendicular phlegmon while gangrenous appendix and appendicular abscess with perforation of the appendix was seen in 26% and 18% of patients, respectively [25]. Early surgery prevents the need for re-hospitalisation, is safe, saves time, is less expensive and promotes an early return to work [24].

The limitations of the study are a single centre study, with smaller sample size.

## Conclusions

Early appendectomy in appendicular mass is safe due to the improvements in surgical techniques and better postoperative care. Prolonged hospital stay was seen in patients of appendicular lump managed conservatively as compared to patients undergoing early exploration. As per this study, early surgical exploration is safe, helps in confirming the diagnosis, shortens the convalescence, prevents the requirement for rehospitalisation, is curative, time-saving, more economical and also reduces hospital stay enabling a quick return to work.
